# Gastrointestinal functions after laparoscopic right colectomy with intracorporeal anastomosis: a pilot randomized clinical trial on effects of abdominal drain, prolonged antibiotic prophylaxis, and D3 lymphadenectomy with complete mesocolic excision

**DOI:** 10.1007/s00384-024-04657-0

**Published:** 2024-07-06

**Authors:** Giuseppe S. Sica, Leandro Siragusa, Brunella Maria Pirozzi, Roberto Sorge, Giorgia Baldini, Cristina Fiorani, Andrea Martina Guida, Vittoria Bellato, Marzia Franceschilli

**Affiliations:** 1 Department of Surgical Sciences, University of Rome “Tor Vergata”, Viale Oxford 81, 00133 Rome, Italy; 2 Department of Biostatistics, University of Rome “Tor Vergata”, Rome, Italy

**Keywords:** Colonic neoplasms, Colectomy, Anastomosis, Mesocolon, Ileus

## Abstract

**Purpose:**

Routine use of abdominal drain or prolonged antibiotic prophylaxis is no longer part of current clinical practice in colorectal surgery. Nevertheless, in patients undergoing laparoscopic right hemicolectomy with intracorporeal anastomosis (ICA), it may reduce perioperative abdominal contamination. Furthermore, in cancer patients, prolonged surgery with extensive dissection such as central vascular ligation and complete mesocolon excision with D3 lymphadenectomy (altogether radical right colectomy RRC) is called responsible for affecting postoperative ileus. The aim was to evaluate postoperative resumption of gastrointestinal functions in patients undergoing right hemicolectomy for cancer with ICA and standard D2 dissection or RRC, with or without abdominal drain and prolonged antibiotic prophylaxis.

**Methods:**

Monocentric factorial parallel arm randomized pilot trial including all consecutive patients undergoing laparoscopic right hemicolectomy and ICA for cancer, in 20 months. Patients were randomized on a 1:1:1 ratio to receive abdominal drain, prolonged antibiotic prophylaxis or neither (I level), and 1:1 to receive RRC or D2 colectomy (II level). Patients were not blinded. The primary aim was the resumption of gastrointestinal functions (time to first gas and stool, time to tolerated fluids and food). Secondary aims were length of stay and complications’ rate. ClinicalTrials.gov no. NCT04977882.

**Results:**

Fifty-seven patients were screened; according to sample size, 36 were randomized, 12 for each arm for postoperative management, and 18 for each arm according to surgical techniques. A difference in time to solid diet favored the group without drain or antibiotic independently from standard or RRC. Furthermore, when patients were divided with respect to surgical technique and into matched cohorts, no differences were seen for primary and secondary outcomes.

**Conclusion:**

Abdominal drainage and prolonged antibiotic prophylaxis in patients undergoing right hemicolectomy for cancer with ICA seem to negatively affect the resumption of a solid diet after laparoscopic right hemicolectomy with ICA for cancer. RRC does not seem to influence gastrointestinal function recovery.

**Supplementary Information:**

The online version contains supplementary material available at 10.1007/s00384-024-04657-0.

## Introduction

Since the standardization of minimally invasive abdominal surgery, right colon cancer surgery has seen two major breakthroughs: the integration of intracorporeal anastomosis (ICA) and the need to standardize the technique for a radical colectomy with complete mesocolic excision, central vascular ligation, and D3 lymphadenectomy, altogether radical right colectomy (RRC) [1].

ICA in laparoscopic right hemicolectomy is associated with reduced short-term morbidity and decreased length of hospital stay suggesting faster recovery [2, 3]. However, the potential contamination of the abdominal cavity during the anastomosis, combined with the prolonged operative time, has been reported to increase the postoperative levels of inflammatory markers, contributing to a delayed recovery process, increased postoperative ileus, and prolonged LOS [4–6]. Furthermore, the extensive surgical dissection and lymphadenectomy to achieve a RRC is also called responsible for prolonged postoperative ileus and LOS [7–11].

The recent standardization of minimally invasive surgery and enhanced recovery pathways protocols has led to changes in some perioperative clinical dogmas. Routine use of abdominal drainage and prolonged antibiotic prophylaxis following colon surgery has been largely abandoned due to the high level of evidence demonstrating a lack of effectiveness in preventing complications such as surgical site infection (SSI) and anastomotic leak or leak sequelae [12–14]. Nevertheless, no studies have been performed to evaluate the role of prophylactic drains and/or antibiotics, after RRC with ICA, on the resumption of gastrointestinal functions.

The aim of this pilot study was to evaluate if abdominal drain, prolonged antibiotic prophylaxis, and RRC affect postoperative resumption of gastrointestinal functions in patients undergoing laparoscopic right colectomy with ICA for cancer.

## Materials and methods

### Trial design

Monocentric, two-level factorial, parallel-arm, pilot randomized clinical trial, conducted from October 2020 to August 2022 comparing patients undergoing laparoscopic right hemicolectomy with ICA for right colon cancer in a single unit of a teaching hospital: Minimally Invasive Surgery Unit, Department of Surgical Sciences, Policlinico Tor Vergata, Rome, Italy.

Patients were initially randomized for postoperative management into three arms to receive prolonged antibiotic prophylaxis (ABX group), abdominal drain placement (DRAIN group), or neither (NONE group) (I level randomization). The same patients were further randomized for surgical technique in two arms to receive RRC (RRC group) or standard hemicolectomy with D2 dissection (STANDARD group) (II level of randomization). Consolidate Standards of Reporting Trials (CONSORT) guidelines for the pilot study were followed [15].

The study was approved by the local IRB of Policlinico Tor Vergata (Rome, Italy), conformed with international ethical recommendations of Helsinki Declarations, and registered at ClinicalTrials.gov no. NCT04977882.

### Population of the study

The study population consisted of all adult patients with right colon cancer scheduled for laparoscopic right hemicolectomy with ICA.

The exclusion criteria were as follows: age > 85 years, BMI > 30, ASA IV, use of systemic steroids, pregnancy, an ongoing infectious disease requiring treatment, inflammatory bowel diseases, history of cancer, T1, T4b, or M1 at cTNM, preoperative radiotherapy, emergency surgery, multivisceral resection, unplanned stoma, and conversion to open approach. T1 patients were excluded because RRC would have represented an overtreatment. T4b patients were excluded because multivisceral resection could have represented a bias in the outcome’s analysis.

Inclusion and exclusion criteria are reported in Table 1.

**Table 1:** Inclusion and exclusion criteria

**Inclusion criteria**	**Exclusion criteria**
Age > 18 years old Right-sided colon cancer Elective setting Laparoscopic approach Right hemicolectomy with intracorporeal anastomosis Informed consent	Age > 85 years old BMI > 30 ASA IV Use of systemic steroids Pregnancy Ongoing infectious disease Inflammatory bowel diseases History of cancer T1 or T4b stage at cTNM Metastatic disease at cTNM Preoperative radiotherapy Emergency surgery Multivisceral resection Unplanned stoma Conversion to open

*BMI* body mass index, *ASA* American Society of Anesthesiologists

### Enrollment, randomization, and blinding

Investigators identified eligible patients through referrals from general practitioners, outpatients’ clinic, oncology unit, and emergency department. Eligible patients provided written informed consent before any study-related intervention. They were randomized on a 1:1:1 ratio to abdominal drain, prolonged antibiotic prophylaxis or neither (I level), and 1:1 to receive RRC or STANDARD colectomy (II level). Randomization was performed by a local investigator via Study Randomizer (2017), a web-based randomization service [16]. Groups were stratified for age and ASA score through the same randomization service to avoid possible disproportion between arms. The allocation sequence was generated with the research manager, and this was concealed from the trained local investigators performing randomization. Investigators and participants were not blinded for group assignment. The enrolment was terminated at the completion of the expected sample size for analysis.

Perioperative management was modulated according to Enhanced Recovery After Surgery (ERAS^®^) Society recommendations [17].

### Perioperative management

All patients in the study were treated according to the same protocol with particular attention to the prevention of postoperative nausea and vomiting (PONV) [18]. For each patient, diagnosis and treatment plan were confirmed during multidisciplinary team discussion. All patients received 5 days of preoperatively immunonutrition with Impact Oral^®^ (Nestlé Health Science, Vevey, Switzerland) three times per day, mechanical bowel preparation, antibiotic prophylaxis with two doses of oral Ciprofloxacin 500 mg, and three doses of Metronidazole 250 mg the day before surgery, and 25 g of maltodextrins in 400 ml of water 2 to 4 h before surgery. At the induction of the anesthesia, 2 g of Cefazolin was also infused.

Only patients in the ABX arm received prolonged intravenous antibiotic prophylaxis as described, in accordance with our Infective Disease Unit. Early mobilization and initiation of oral fluids were implemented a few hours after surgery whenever possible. If water and tea were well tolerated, patients were allowed to progress to a soft diet already on the first postoperative day (POD). White blood cells (WBC) and C-reactive protein (CRP) were checked on POD 1 and repeated on POD 3 when it was also checked the level of serum procalcitonin (PCT). In POD 3, in case of CRP level > 150 mg/L or for an increase > 50 mg/L (from the baseline on POD 1), patients were CT-scanned, and a step back on oral intake was undertaken. Hospital stay was prolonged in most of these patients and CRP and PCT were checked again in POD 5 before discharge. Abdominal drains were removed within 36 h from surgery.

### Surgical technique

#### Laparoscopic standard D2 right hemicolectomy (STANDARD)

The operation is conducted with patients in the supine position and pneumoperitoneum at 12 mm-Hg induced through an optical trocar in the left flank; three operative trocars are subsequently placed under direct vision. A medial-to-lateral surgical dissection and high tie of the ileocolic vessels (IC) are undertaken without dissecting the anterior surface of the superior mesenteric vein (SMV). The gastro-colic trunk of Henle (GCTH) is not isolated; the right colic vein (when present) and the right branches of the middle colic vessels are taken more peripherical, during the division of the transverse mesocolon. After the surgical resection, the ileal and colic stumps are stapled with a single fire of a 60-mm mechanical stapler in an isoperistaltic fashion and the common enterotomy closed with double running barbed 3-0 sutures. The mesentery is closed with polymer ligating clips as previously described [19], and the specimen is extracted through a wound protector after enlarging the port incision in the right inguinal region.

#### Laparoscopic radical right colectomy with CME and D3 lymphadenectomy (RRC)

The operation is conducted with the same installation and trocars position of STANDARD, but the dissection starts over the landmark given by SMV. The SMV is freed anteriorly and on its right-hand side from all the lympho-adipose tissue. Once the SMV is fully exposed, the IC vessels are dissected and divided at the junction with the efferent vessels. The dissection moves upward along the same dissection line to identify the right colic vein and the GCTH. No medial to later dissection is carried out until the SMV is completely exposed before reaching the uncinate process of the pancreas. At this point, the veins to the right colon are divided, but the gastroepiploic vein and artery are preserved unless the tumor is located at the hepatic flexure. The divided mesentery is lifted and tilted to the right, and the medial-to-later dissection starts following the embryological plane over Fredet’s fascia. The right branches of the middle colic vessels are divided, and the mesocolon is divided on the left side of the middle colic artery. The anastomoses are fashioned intracorporeally as described in STANDARD.

A 19 Fr abdominal drain was left in the right paracolic gutter only in patients in the DRAIN arm.

All the operations for this trial were performed in a single unit.

### Outcome measures

The outcome measures were analyzed based on the two levels of randomization: level I compares the intervention groups NONE vs DRAIN vs ABX, and level II compares the level of randomization between RRC vs STANDARD. By analyzing the outcomes independently at both levels of randomization, the study aimed to examine the effects of each intervention (DRAIN vs ABX) and surgical technique (RRC vs STANDARD) on the measured outcomes. The same analysis was performed for the six-cohort determined by matching the two levels of randomization.

The primary outcome was to evaluate postoperative gastrointestinal functions. In this study, impairment of gastrointestinal functions was considered equivalent to postoperative ileus, defined as the absence of peristalsis that hindered the progression of the patient’s refeeding or required a regression to fasting in the refeeding process [20]. For the analysis of results, postoperative gastrointestinal functions included time to first gas and stool and time to tolerated fluids (clear fluids) and food (soft diet).

Secondary outcomes included a 30-day rate of complications. Complications were divided into general (graded according to Clavien-Dindo classification) and specific complications such as anastomotic leak (defined as a defect of the intestinal wall at the anastomotic site leading to a communication between the intra- and extraluminal compartments), SSI defined according to the Center for Disease Control and Prevention, CDC/NHNS), ileus, and bleeding [21–23]. Secondary outcomes also included LOS and prolonged LOS (pLOS), defined as any LOS greater than 1.5 times the median LOS of the whole sample. Other measured variables were as follows: time to mobilization; incidence of postoperative PONV and need for transfusions; 30-day readmission, reintervention, and mortality rate; mean WBC (10^3^/μL) at POD 1 and 3, CRP (mg/L) at POD 1, 3, and 5, and PCT l (mg/L) at POD 3 and 5.

### Sample size

Sample size calculation was based on the mean time to tolerated food in the historical population of patients undergoing laparoscopic right hemicolectomy in the same unit (2.3 ± 1.1 days) [24]. A decrease in the mean time to tolerated food of 30% was considered clinically meaningful in sample size calculation. Using an *α* of 0.05, power of 0.80 (one-sided Wilcoxon test), and expecting a dropout rate of 10%, an estimated sample size of 36 patients was aimed for (12 per arm). This sample size was sufficient to demonstrate a difference in the primary outcome for the first and second levels of randomization. For matched cohorts, an estimated sample size of 72 patients could demonstrate a difference in the primary outcome.

### Statistical analysis

For each patient, clinical data were entered in a specific case report form (CRF) and subsequently exported on an Excel spreadsheet (Microsoft, Redmond, WA, USA). Statistical analysis was then conducted using the social science statistical package for Windows, version 15.0 (SPSS, Chicago, IL, USA).

All normally distributed parameters (confirmed by histograms and Kolmogorov-Smirnov tests) are presented as mean ± standard deviation, while non-normally distributed parameters are presented as median and range (min; max). Frequency occurrence parameters are described as percentages. Comparisons between groups of normally distributed variables were conducted using *T*-student or one-way ANOVA, while categorical occurrence comparisons were performed using the chi-square test or Fisher’s exact test. A *p*-value < 0.05 was considered statistically significant.

## Results

### Study population

From October 2020 to August 2022, 57 patients diagnosed with right colon cancer, scheduled for a right hemicolectomy with ICA, were assessed for eligibility. Twenty-one patients were excluded for different reasons: two T1, two T4b, and five M1 at cTNM, three with BMI > 30, three over 85-year-old, two emergency procedure, two patients did not consent, one ASA IV, one with synchronous neoplasia.

Thirty-six patients, according to the calculated sample size, were enrolled and randomized for intervention in three groups for the first level of randomization: 12 receiving prolonged antibiotic prophylaxis (ABX group), 12 abdominal drain placement (DRAIN group), and 12 none of them (NONE group).

The same patients were also randomized according to a surgical technique for the second level of randomization: 18 receiving RRC (RRC group) and 18 receiving standard D2 oncological hemicolectomy (STANDARD group).

Patients were finally divided into six groups according to the randomization received for intervention and surgical technique: six NONE-RRC, six NONE-STANDARD, three DRAIN-RRC, nine DRAIN-STANDARD, nine ABX-RRC, three ABX-STANDARD.

No patient was lost at follow-up; 36 patients were included in the final analysis.

Most of this study was conducted during the SARS-CoV-2 pandemic. There were no contraindications, nor specific indications from local IRB, but the Covid test for all the patients. Enrollment was slower though, and the study terminated in August 2022, having reached the intended sample size of 36 patients for the I and II level of randomization.

**Fig. 1:**
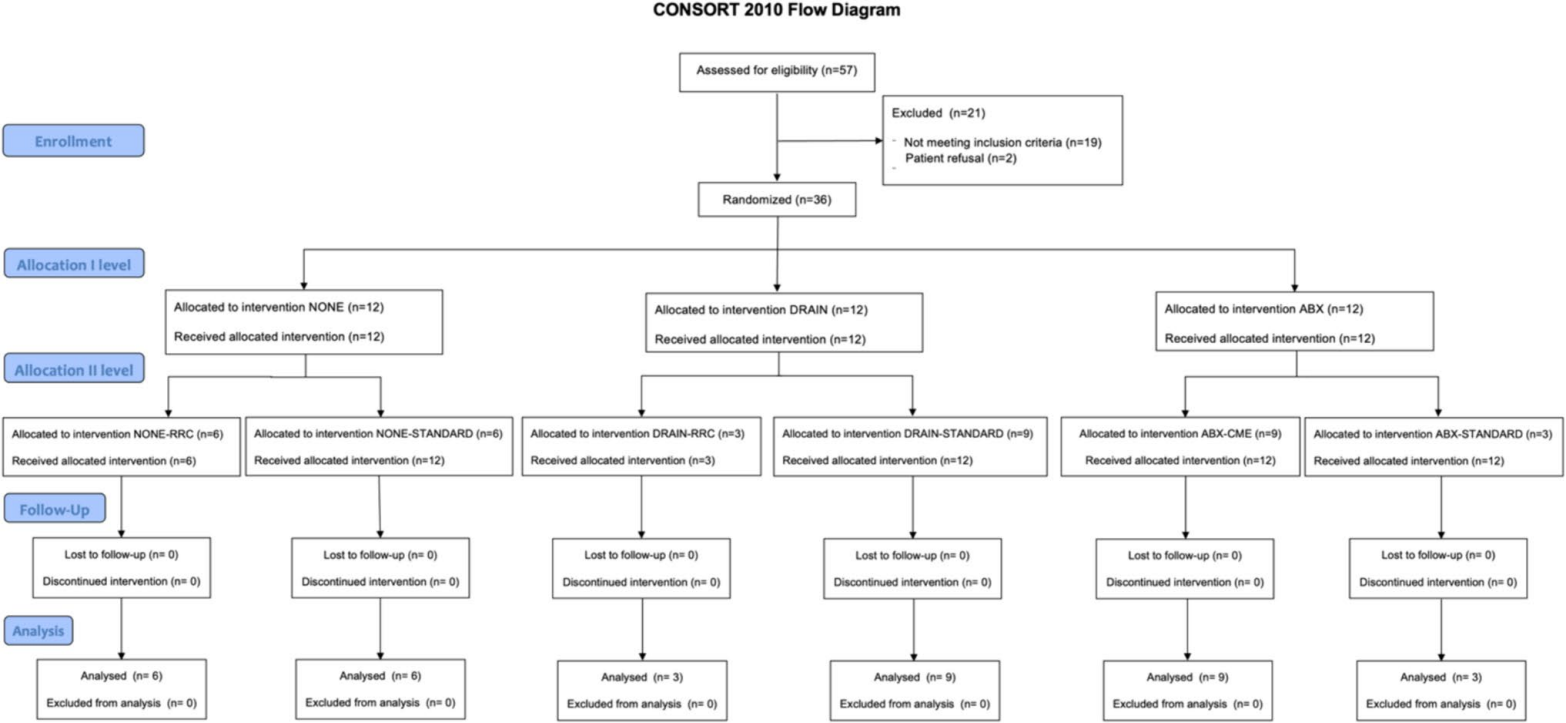
CONSORT flow diagram

The CONSORT flow diagram is displayed in Fig. 1.

### I level randomization (NONE vs DRAIN vs ABX)

#### Baseline characteristics

Groups were comparable in terms of age, preoperative BMI, ASA score, previous abdominal surgery, smoke and alcohol use, comorbidities (hypertension, cardiac, diabetes, respiratory), CCI, preoperative hemoglobin and albumin, cTNM staging, surgical technique, and operative time (*p*-value = ns). The three groups significantly differ in sex distribution (*p*-value = 0.016).

Baseline characteristics of NONE vs DRAIN vs ABX are reported in Table 2.

**Table 2:** Baseline characteristics NONE vs DRAIN vs ABX

**Parameters**	**NONE (*n* = 12)**	**DRAIN (*n* = 12)**	**ABX (*n* = 12)**	***p***
Age (mean, SD) (year)	72.3 ± 8.8	72.1 ± 9.0	68.2 ± .16.4	0.642
Preoperative BMI (mean, SD)	26.1 ± 3.5	26.7 ± 3.5	26.1 ± 3.1	0.890
Sex (*n*, %)				**0.016**
Male	3 25%	10 83.3%	6 50%	
Female	9 75%	2 16.7%	6 50%	
ASA score (*n*, %)				0.640
1	0 0%	0 0%	0 0%	
2	7 58.3%	5 41.7%	5 41.7%	
3	5 41.7%	7 58.3%	7 58.3%	
Previous abdominal surgery (*n*, %)	10 83.3%	6 50%	6 50%	0.154
Smoker (*n*, %)				0.853
Yes	1 8.3%	2 16.7%	2 16.7%	
No	6 50%	5 50%	7 58.3%	
Ex	5 41.7%	5 50%	3 25%	
Alcohol abuse (*n*, %)	0 0%	1 8.3%	0 0%	0.358
Comorbidity (*n*, %)				
Hypertension	6 50%	6 50%	7 58.3%	0.895
Cardiac	2 16.7%	4 33.3%	5 41.7%	0.400
Diabetes	0 0%	2 16.7%	3 25%	0.197
Respiratory	3 25%	3 25%	0 0%	0.165
CCI (mean, SD)	5.9 ± 1.2	7.1 ± 1.8	6.1 ± 2.2	0.224
Hb pre-op (mean, SD) g/dL	12.4 ± 2.1	11.5 ± 1.5	11.7 ± 2.2	0.502
Albumin pre-op (mean, SD) g/dL	4.12 ± 0.3	4.39 ± 0.4	4.09 ± 0.5	0.142
TNM staging (*n*, %)				0.616
I	5 41.7%	3 25%	2 16.7%	
II	5 41.7%	8 66.7%	8 66.6%	
III	2 16.6%	1 8.3%	2 16.7%	
Type of surgery (*n*, %)				0.052
RRC	6 50%	3 25%	9 75%	
STANDARD	6 50%	9 75%	3 25%	
Operative time (mean, SD)	162.9 ± 20.3	164.3 ± 40.1	162.3 ± 45.2	0.992

*ASA* American Society of Anesthesiologists, *CCI* Carlson Comorbidity index, *Hb* hemoglobin, *RRC* radical right colectomy

#### Primary and secondary outcomes

**Fig. 2:**
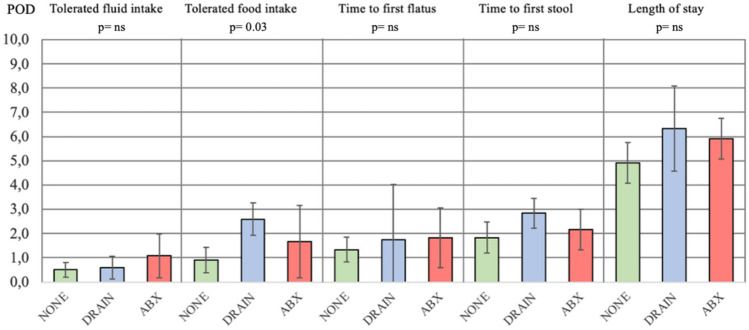
Gastrointestinal functions resumption outcomes of NONE vs DRAIN vs ABX

For what concern the primary outcome, results were comparable between the three groups NONE, DRAIN, and ABX: time to tolerated fluid intake respectively 0.5 vs 0.6 vs 1.1 days, first flatus 1.3 vs 1.8 vs 1.8 days; first stool 1.8 vs 2.8 vs 2.2 days (*p*-value = ns). Nevertheless, the mean time to tolerated food intake was shorter for the NONE group with respect to both DRAIN and ABX groups (respectively 0.9 days vs 2.6 vs 1.7; *p*-value = 0.038) (Fig. 2).

Both mean LOS and median pLOS were comparable among groups (respectively 4.9 vs 6.3 vs 5.9 days, and 2 vs 6, vs 3 days). No differences were found in the number of general and specific postoperative complications, number of reinterventions, inflammatory markers levels (WBC, CRP, PCT), readmission’s rate, and 30-day mortality.

Primary and secondary outcomes of NONE vs DRAIN vs ABX are reported in Table 3.

**Table 3:** Primary and secondary outcomes of NONE vs DRAIN vs ABX

**Parameters**	**NONE (*n* = 12)**	**DRAIN (*n* = 12)**	**ABX (*n* = 12)**	***p***
Mobilization (mean, SD) POD	0.8 ± 0.5	1 ± 0.9	0.8 ± 0.4	0.589
Tolerated fluid intake (mean, SD) POD	0.5 ± 0.5	0.6 ± 0.7	1.1 ± 1.5	0.314
Tolerated food intake (mean, SD) POD	0.9 ± 0.5	2.6 ± 2.3	1.7 ± 1.2	**0.038**
Time to first flatus (mean, SD) POD	1.3 ± 0.7	1.8 ± 0.6	1.8 ± 0.8	0.196
Time to first stool (mean, SD) POD	1.8 ± 0.8	2.8 ± 1.7	2.2 ± 0.8	0.139
PONV (*n*, %)	1 8.3%	1 8.3%	4 33.3%	0.141
LOS (mean, SD) days	4.9 ± 2.8	6.3 ± 2.5	5.9 ± 3.8	0.512
pLOS (median 4.5) (*n*, %)	2 16.7%	6 50%	3 27.3%	0.200
Complications (*n*, %)	3 25%	5 41.7%	4 33.3%	0.687
SSI (*n*, %)	0 0%	1 8.3%	0 0%	0.358
Anastomotic leak (*n*, %)	0 0%	2 16.7%	1 8.3%	0.336
Anastomotic leak requiring reintervention (*n*, %)	0 0%	0 0%	1 8.3%	0.358
Bleeding (*n*, %)	0 0%	2 16.7%	2 16.7%	0.169
Transfusion (*n*, %)	3 25%	3 25%	5 41.7%	0.592
Ileus (*n*, %)	2 16.7%	2 16.7%	2 16.7%	1.000
Clavien-Dindo (*n*, %)				**0.094**
0	4 33.3%	1 8.3%	6 50%	
1	3 25%	5 41.7%	0 0%	
2	4 33.3%	6 50%	2 16.7%	
3	0 0%	0 0%	1 8.3%	
4	1 8.3%	0 0%	2 16.7%	
5	0 0%	0 0%	1 8.3%	
Readmission (*n*, %)	0 0%	0 0%	1 8.3%	0.358
Reintervention (*n*, %)	1 8.3%	0 0%	3 25%	0.140
30-day mortality (*n*, %)	0 0%	0 0%	1 8.3%	0.358
WBC (mean, SD) 10^3^/μL				
POD I	14.32 ± 15,9	10.3 ± 5.3	10.4 ± 2.1	0.530
POD III	7.8 ± 3.1	7 ± 1.4	9.2 ± 4.1	0.260
CRP (mean, SD) mg/L				
POD I	60.5 ± 36.7	66.1 ± 37.6	56.5 ± 30	0.795
POD III	116.8 ± 74.6	152.7 ± 96.5	124.6 ± 102	0.659
POD V	96.8 ± 57.4	107.1 ± 69.3	120.4 ± 80.6	0.864
PCT (mean, SD) ng/mL				
POD III	0.45 ± 0.58	1.03 ± 1.8	1.15 ± 1.95	0.535
POD V	1.2 ± 1.41	0.33 ± 0.46	2.64 ± 3.62	0.241

*POD* post-operative day, *CV* urinary catheter, *PONV* postoperative nausea and vomiting, *LOS* length of stay, *pLOS* prolonged length of stay, *SSI* surgical site infection, *WCC* white cell count, *CRP* C-reactive protein, *PCT* procalcitonin

### II level randomization RRC vs STANDARD

#### Baseline characteristics

No differences in patients’ baseline characteristics were found, but the preoperative BMI differed significantly between the RRC and STANDARD groups (27.7 vs 24.9 respectively; *p*-value = 0.01).

Baseline characteristics of RRC vs STANDARD groups are shown in Table 4.

**Table 4:** Baseline characteristics of STANDARD vs RRC

**Parameters**	**STANDARD (*n* = 18)**	**RRC (*n* = 18)**	***p***
Age (mean years, SD)	73 ± 8.1	68.7 ± 14.5	0.275
Preoperative BMI (mean kg\m^2^, SD)	27.7 ± 3.8	24.9 ± 1.9	**0.010**
Sex (*n*, %)			0.738
Male	10 55.6%	9 50%	
Female	8 44.4%	9 50%	
ASA score (*n*, %)			0.738
1	0 0%	0 0%	
2	8 44.4%	9 50%	
3	10 55.6%	9 50%	
Prev. abdominal surg. (*n*, %)	12 66.7%	10 55.6%	0.494
Smoker (*n*, %)			0.410
Yes	3 16.7%	2 11.1%	
No	7 38.9%	11 61.1%	
Ex	8 44.4%	5 27.8%	
Alcohol (*n*, %)	1 5.6%	0 0%	0.310
Comorbidity (*n*, %)			
Hypertension	12 66.7%	7 38.9%	0.095
Cardiac	6 33.3%	5 27.8%	0.717
Diabetes	1 5.6%	4 22.2%	0.148
Respiratory	5 27.8%	1 5.6%	0.074
CCI (mean, SD)	6.8 ± 1.6	5.9 ± 1.8	0.112
Hb pre-op (mean, SD) g/dL	12 ± 1.9	11.8 ± 2	0.755
Albumin pre-op (mean, SD) g/dL	4.24 ± 0.4	4.17 ± 0.42	0.620
Operative time (mean, SD)	168.6 ± 31.7	157.7 ± 38.7	0.369
TNM staging (*n*, %)			0.325
I	6 33.3%	4 22.2%	
II	11 61.1%	10 55.6%	
III	1 5.6%	4 22.2%	

*ASA* American Society of Anesthesiologists, *CCI* Charlson Comorbidity index, *Hb* hemoglobin, *RRC* radical right colectomy

#### Primary and secondary outcomes

**Fig. 3:**
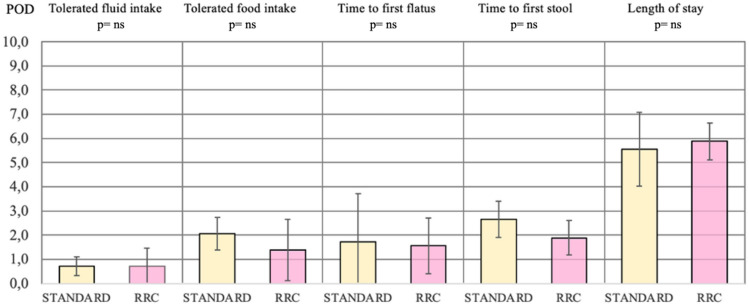
Gastrointestinal functions resumption outcomes of STANDARD vs RRC

No differences in primary outcomes were found between STANDARD and RRC groups: time to tolerated fluid intake respectively 0.7 vs 0.7 days, time to tolerated food intake 2.1 vs 1.4 days, first flatus 1.7 vs 1.6, first stool 2.7 vs 1.9 days (*p*-value = ns). The average of patients’ LOS and pLOS were comparable (*p*-value = ns) (Fig. 3).

No differences in the number of general and specific complications nor in the number of readmissions and 30-day mortality were found, but the number of reinterventions was statistically higher in the RRC group (22.2% vs 0%; *p*-value = 0.034). The level of inflammatory markers (WBC, CRP, PCT) did not vary among groups.

The primary and secondary outcomes of RRC vs STANDARD are summarized in Table 5.

**Table 5:** Results of primary and secondary outcomes of STANDARD vs RRC

**Parameters**	**STANDARD (*n* = 18)**	**RRC (*n* = 18)**	***p***
Mobilization (mean, SD) POD	0.8 ± 0.8	0.9 ± 0.3	0.783
Tolerated fluid intake (mean, SD) POD	0.7 ± 0.7	0.7 ± 1.3	1.000
Tolerated food intake (mean, SD) POD	2.1 ± 2.0	1.4 ± 1.1	0.225
Time to first flatus (mean, SD) POD	1.7 ± 0.8	1.6 ± 0.7	0.497
Time to first stool (mean, SD) POD	2.7 ± 1.5	1.9 ± 0.8	0.062
PONV (*n*, %)	1 5.6%	5 27.8%	0.100
LOS (mean, SD) days	5.6 ± 2.2	5.9 ± 3.7	0.754
pLOS (median 4.5) (*n*, %)	5 27.8%	6 33.3%	0.632
Complications (*n*, %)	4 22.2%	8 44.4%	0.157
SSI (*n*, %)	0 0%	1 5.6%	0.310
Anastomotic leak (*n*, %)	2 11.1%	1 5.6%	0.546
Anastomotic leak requiring reintervention (*n*, %)	0 0%	1 5.6%	0.310
Bleeding (*n*, %)	3 16.7%	1 5.6%	0.289
Transfusion (*n*, %)	4 22.2%	7 38.9%	0.278
Ileus (*n*, %)	2 11.1%	4 22.2%	0.371
Clavien-Dindo (*n*, %)			0.226
0	5 27.8%	6 33.3%	
1	5 27.8%	3 16.7%	
2	8 44.4%	4 22.2%	
3	0 0%	1 5.6%	
4	0 0%	3 16.7%	
5	0 0%	1 5.6%	
Readmission (*n*, %)	0 0%	1 5.6%	0.310
Reintervention (*n*, %)	0 0%	4 22.2%	**0.034**
30-days mortality (*n*, %)	0 0%	1 5.6%	0.310
Hb loss (mean, SD)	1.8 ± 1.1	1.5 ± 0.7	0.222
WBC (mean, SD) 10^3^/μL			
POD I	13.1 ± 13.6	10.3 ± 2.3	0.408
POD III	7.3 ± 2.2	8.7 ± 3.7	0.174
CRP (mean, SD) mg/L			
POD I	58.6 ± 33.1	63.6 ± 35.9	0.666
POD III	128.1 ± 81.5	130.6 ± 99.2	0.937
POD V	97 ± 57.1	124.9 ± 80	0.404
PCT (mean, SD) ng/mL			
POD III	0.84 ± 1.38	0.91 ± 1.75	0.893
POD V	0.74 ± 0.92	2.05 ± 3.34	0.300

*POD* postoperative day, *CV* urinary catheter, *NGT* naso-gastric tube, *LOS* length of stay, *pLOS* prolonged length of stay, *SSI* surgical site infection, *WBC* white blood count, *CRP* C-reactive protein, *PCT* procalcitonin

### Matched cohorts (STANDARD-NONE, STANDARD-DRAIN, STANDARD-ABX, RRC-NONE, RRC-DRAIN, RRC-ABX)

#### Baseline characteristics

The patients’ baseline characteristics between all the six groups did not differ but the sex distribution showed some variability with statistical significance.

The baseline characteristics are summarized in Table S1.

#### Primary and secondary outcomes

The mean time to mobilization, to tolerated fluids and food intake, to first flatus, and to first stool were also similar between the two groups (*p*-value = ns). The patients’ LOS and pLOS were similar (*p*-value = ns). No differences in general and specific complications were found; the anastomotic leak rate and bleeding rate were also similar, but the rate of SSI was noted higher in group RRC-DRAIN (*p*-value = 0.045).

The primary and secondary results are summarized in Table S2.

## Discussion

This study examines if abdominal drains or prolonged antibiotic prophylaxis prolong postoperative ileus and cause a delay in gastrointestinal functions’ resumption after standard laparoscopic right hemicolectomy and RRC with ICA.

Prophylactic drain and antibiotics were found unnecessary in patients undergoing standard right hemicolectomy or RRC with ICA, with respect to the postoperative resumption of gastrointestinal functions. In fact, time to solid diet favored the group without drain or antibiotics in both groups. When patients were divided into matched cohorts, no differences were seen for primary and secondary outcomes.

The rationale of this study was the observation that RRC with ICA carry a potential higher risk of determining local abdominal infections due to the prolonged exposure to intestinal bacteria combined with larger fluids extravasations [5]. As a matter of fact, both these phenomena are incontrovertible: ICA during minimally invasive surgery is a procedure that converts a clean procedure into a contaminated one; RRC requires a wider anatomical and nodal dissection and increases postoperative fluid extravasation. Indeed, several authors, during the first experiences of RRC, insisted on evaluating the resumption of gastrointestinal functions after this extensive radical procedure [25, 26]. The reason is the perceived prolonged postoperative ileus due to the abovementioned factors. Some authors gave other explanations, such as injuries to the superior mesenteric nerve plexus [27, 28]. Probably, the prolonged ileus during RRC was a phenomenon found during preliminary experiences, when things might have been overlooked and numbers were small to draw final conclusions.

To our knowledge, this is the first study that examined the combined effect of ICA within the context of laparoscopic RRC.

The factorial design of this study, with two levels of intervention, was chosen to assess both the independent effects of perioperative interventions and surgical techniques and their potential combined effects.

Postoperative ileus is a common feature following colectomy, sometimes a complication. It seems to occur more frequently after right hemicolectomy compared to left-sided colectomies, with an incidence ranging between 3 and 30% [29, 30]. Several meta-analyses and randomized trials have been conducted to evaluate postoperative ileus after right hemicolectomy for cancer with intracorporeal versus extracorporeal anastomosis [2, 31–34]. In the meta-analysis of randomized trials, Zhang et al. reported a lower incidence of ileus in patients having ICA (4.5%), with a relative risk of 0.62 [2]. Results from another meta-analysis showed that ICA resulted in a shorter time to first flatus and first defecation, and an incidence of ileus of 7.1% [31]. A randomized trial by Malczak et al. comparing intracorporeal and extracorporeal anastomosis found no difference in time to first gas, LOS, and ileus rate (5%) but found a difference in time to first stool [32]. A recent multicentric prospective four-cohort study (laparoscopic extracorporeal and ICA; robotic extracorporeal and ICA) demonstrated lower overall complication rates after ICA, specifically less ileus, and PONV after robot-assisted procedures [33]. However, it is possible that differences in reporting postoperative ileus and differences in the definitions could make a comparison between studies difficult [35, 36]. Furthermore, recent evidence suggested that the common assumption of postoperative paralysis might be incorrect and that the distal colon becomes hyperactive following surgery [37]. The post-operative inflammatory response is important in the pathophysiology of ileus, but the time course of this in humans remains unclear, with most of the evidence coming from animal models [38].

In this study, the primary outcome was to evaluate postoperative gastrointestinal functions, considered equivalent to postoperative ileus, and defined as the absence of bowel movement that hindered the progression of the patient’s refeeding or required a regression to fasting in the refeeding process [20]. However, for the analysis of results, postoperative gastrointestinal functions included time to first bowel movement, gas and stool, and time to tolerated clear fluids and soft diet.

Regarding the first level of randomization and about the primary outcome, the rate of postoperative ileus in the three groups did not vary and was in line with that reported in the literature for laparoscopic right hemicolectomy with ICA, despite the extent of surgical dissection (STANDARD vs RRC) [1, 4, 31, 32]. Nevertheless, patients in the NONE group had a significant shorter time to tolerate food, with an average of 0.9 days, compared to 1.7 days in the ABX group and 2.6 days in the DRAIN group. Another difference, although not statistically significant, is in the time to tolerated fluid: 0.5 days in the NONE group and 0.6 days in the DRAIN Group, compared to 1.1 days in the ABX group. A possible explanation for this result is that the presence of drains may cause discomfort and hinder patients’ mobility; it might also contribute to increase patients’ anxiety and fear of accidents, thus reducing motility further, and compliance to protocols of fast-track recovery [14, 17]. Regarding prolonged antibiotic prophylaxis, the need for multiple infusions may contribute to reducing postoperative mobilization and can also increase PONV, ultimately leading to slower postoperative re-alimentation [13, 39]. Of note, one-third of patients in the ABX arm had prolonged PONV; this finding, even if not statistically significant, provides additional support for the potential role of antibiotics in slower resumption of gastrointestinal functions.

Multiple studies have highlighted the limited benefits, if any, of drainage placement and prolonged antibiotic prophylaxis after colorectal surgery in terms of reductions of general complications, such as ileus, anastomotic leaks, wound infections, and mortality [12, 14, 40–44]. Drains are not recommended by ERAS^®^ guidelines, and this study confirmed that they are to be avoided also in RRC with ICA [17].

Concerning secondary outcomes, there were no significant differences in LOS and pLOS, but LOS was slightly shorter in the NONE group. The incidence of general and specific (surgical site infections, anastomotic leaks, bleeding) complications, transfusions, 30-day readmission, reoperation, and mortality also did not show statistically significant differences and was in line with the literature [2–4].

Interestingly, the levels of inflammatory markers, such as CRP and PCT, did not differ among groups, confirming that the idea of reducing abdominal infections in patients undergoing laparoscopic ICA, by means of abdominal drain or prolonged antibiotic prophylaxis, is probably not true.

Regarding the second level of randomization, the comparison between RRC and standard D2 hemicolectomy in this study did not show any significant differences in postoperative gastrointestinal functions, nor in LOS, pLOS, general and specific complications, need for blood transfusions, readmission rates, and 30-day mortality. The higher rate of reintervention in the RRC group could be attributed to the cautious approach taken at the beginning of the RRC’s experience. Indeed, among the four reoperations, three were exploratory laparoscopy.

The matched cohort analysis also did not show any significant differences in primary outcomes between the interventions combined with the surgical technique. This suggests that each intervention is safe and feasible for both patients’ subsets.

This study has some limitations, and its findings need to be taken with caution. Firstly, the sample size is powered to detect a difference in the two levels of randomization but not for the matched cohorts, for which the small sample size may limit the generalizability of the results. In fact, the factorial design aims to assess the interaction between interventions, but with small matched cohorts, there are little data to evaluate these interactions, increasing the risk of type II errors. The second limitation is in the study design, with two levels of randomization, which introduces the possibility of confounding bias between the two interventions. Also, exclusion criteria may reduce result generalization, and being a single-center study, there is a possibility of selection bias. Other possible biases may be the differences in BMI between RRC and STANDARD groups and gender between NONE, DRAIN, and ABX groups. However, obese patients were excluded from the study, and in the literature, there is no evidence regarding additional risks in ileus, complications, and LOS in overweight patients, nor differences among different sexes.

Nevertheless, these limitations are justified considering the nature of a pilot study on a novel search, being its primary purpose simply that assess the feasibility of the interventions (no drain; no prolonged antibiotic prophylaxis) during RRC with ICA. Furthermore, the study’s comprehensive assessment of postoperative outcomes and internal validity due to the single-center setting might enhance its significance; in fact, small studies can make surrogate markers when examining associations, and it is often better to test a new research hypothesis in a small number of subjects first.

Additional prospective studies with larger samples would be required to validate these results and to investigate the recovery of gastrointestinal functions in the matched cohorts. However, with robotic surgery overtaking over laparoscopic surgery, many more ICA and RRC will be performed worldwide. The precision of the robotic-assisted surgery should further implement and possibly confirm present results.

## Conclusion

Abdominal drainage and prolonged antibiotic prophylaxis in a patient undergoing right hemicolectomy for cancer with ICA seem to negatively affect the resumption of a solid diet after laparoscopic right hemicolectomy with ICA for cancer, and their use is discouraged. RRC does not seem to influence gastrointestinal function recovery.

## Supplementary Information

Below is the link to the electronic supplementary material.

Supplementary file1 (DOCX 35 KB)

## Data Availability

No datasets were generated or analyzed during the current study.
