# Supplementary material for: Gastrointestinal functions after laparoscopic right colectomy with intracorporeal anastomosis: a pilot randomized clinical trial on effects of abdominal drain, prolonged antibiotic prophylaxis, and D3 lymphadenectomy with complete mesocolic excision
Source: Int J Colorectal Dis. 2024 Jul 6;39(1):102. doi: 10.1007/s00384-024-04657-0 (PMC11227461; doi:10.1007/s00384-024-04657-0)
Supplement: Supplementary file 1 — Supplementary file1 (DOCX 35 KB) [file 384_2024_4657_MOESM1_ESM.docx]

**Table S1 Baseline characteristics**

| **Parameters** | **STANDARD-NONE**  **(n=6)** | **STANDARD-DRAIN**  **(n=9)** | **STANDARD-ABX**  **(n=3)** | **RRC-NONE**  **(n=6)** | **RRC-DRAIN**  **(n=3)** | **RRC-ABX**  **(n=9)** | **P** |
| --- | --- | --- | --- | --- | --- | --- | --- |
| Age (mean, SD) year | 75.3±7.7 | 74.7±5.5 | 63.3±.10.2 | 69.2±9.5 | 64.3±14.4 | 69.8±18.2 | 0.550 |
| Preoperative BMI  (mean, SD) | 27.11±4.14 | 27.22±3.92 | 30.27±2.68 | 25.18±2.62 | 25.12±1.09 | 24.71±1.75 | 0.112 |
| Sex (n, %)  Male  Female | 2 33.3%  6 66.7% | 8 88.9%  1 11.1% | 0 0%  3 100% | 1 16.7%  5 83.3% | 2 66.7%  1 33.3% | 6 66.7%  3 33.3% | **0.023** |
| ASA score  (n, %)  1  2  3 | 0 0%  2 33.3%  4 66.7% | 0 0%  1 11.1%  8 88.9% | 0 0%  1 33.3%  2 66.7% | 0 0%  6 100%  0 0% | 0 0%  2 66.7%  1 33.3% | 0 0%  4 44.4%  5 55.6% | 0.705 |
| Previous abdominal surgery (n, %) | 5 83.3% | 5 55.6% | 2 66.7% | 5 83.3% | 1 33.3% | 4 44.4% | 0.457 |
| Smoker (n, %)  Yes  No  Ex | 1 16.7%  2 33.3%  3 55.6% | 1 11.1%  3 33.3%  5 55.6% | 1 33.3%  2 66.7%  0 0% | 0 0%  4 66.7%  2 33.3% | 1 33.3%  2 66.7%  0 0% | 1 11.1%  5 55.6%  3 33.3% | 0.666 |
| Alcohol (n, %) | 0 0% | 1 11.1% | 0 0% | 0 0% | 0 0% | 0 0% | 0.687 |
| Comorbidity  (n, %)  Hypertension  Cardiac  Diabetes  Respiratory | 5 83.3%  2 33.3%  0 0%  3 50% | 5 55.6%  3 33.3%  1 11.1%  2 22.2% | 2 66.7%  1 33.3%  0 0%  0 0% | 1 16.7%  0 0%  0 0%  0 0% | 1 33.3%  1 33.3%  1 33.3%  1 33.3% | 5 55.6%  4 44.4%  3 33.3%  0 0% | 0.294  0.618  0.281  0.101 |
| CCI  (mean, SD) | 6.5±1.2 | 7.7±1.5 | 5.0±1.0 | 5.3±0.8 | 5.3±1.5 | 6.4±2.4 | 0.070 |
| Hb pre-op  (mean, SD) | 12±2.5 | 11.5±1.4 | 13.5±1.36 | 12.9±1.57 | 11.6±2.15 | 11.1±2.14 | 0.359 |
| ALB pre-op  (mean, SD) | 4±0.3 | 4.4±0.4 | 4.1±0.3 | 4.2±0.3 | 4.3±0.2 | 4.1±0.6 | 0.448 |
| Staging (n, %)  I  II  III | 2 33.3%  3 50%  1 16.7% | 3 33.3%  6 66.7%  0 0% | 1 33.3%  2 66.7%  0 0% | 3 50%  2 33.3%  1 16.7% | 0 0%  2 66.7%  1 33.3% | 1 11.1%  6 66.7%  2 22.2% | 0.576 |
| Op. time  (mean, SD) | 169.8±22.3 | 174.2±41.5 | 149.3±14 | 156±16.7 | 134.3±12.1 | 166.7±51.8 | 0.607 |

ASA, American Society of Anaesthesiologists; CCI, Charlson Comorbidity index; Hb, hemoglobin.

**Table S2. Results of primary and secondary outcomes**

| **Parameters** | **STANDARD-NONE**  **(n=6)** | **STANDARD-DRAIN**  **(n=9)** | **STANDARD-ABX**  **(n=3)** | **RRC-NONE**  **(n=6)** | **RRC-DRAIN**  **(n=3)** | **RRC-ABX**  **(n=9)** | **P** |
| --- | --- | --- | --- | --- | --- | --- | --- |
| Mobilization (mean, SD)  POD | 0.5±0.5 | 1.1±0.9 | 0.7±0.6 | 1±0.0 | 0.7±0.6 | 0.9±0.3 | 0.459 |
| Tolerated fluid intake (mean, SD)  POD | 0.7±0.5 | 0.7±0.7 | 1±1 | 0.3±0.5 | 0.3±0.6 | 1.1±1.7 | 0.731 |
| Tolerated food intake (mean, SD)  POD | 1±0.6 | 3±2.4 | 1.3±0.6 | 0.8±0.4 | 1.3±1.3 | 1.8±1.4 | 0.104 |
| Time to first flatus  (mean, SD) POD | 1.5±0.8 | 1.8±0.7 | 2±1 | 1.2±0.4 | 1.7±0.6 | 1.8±0.8 | 0.547 |
| Time to first stool (mean, SD) POD | 2±1.1 | 3.2±1.9 | 2.3±0.6 | 1.7±0.5 | 1.7±0.6 | 2.1±0.9 | 0.169 |
| PONV  (n, %) | 0 0% | 1 11.1% | 0 0% | 1 16.7% | 0 0% | 4 44.4% | 0.231 |
| LOS  (mean, SD) | 4.2±1.2 | 6.7±2.6 | 5±1 | 5.7±3.8 | 5.3±2.3 | 6.3±4.5 | 0.736 |
| PLOS (median 4,5) (n, %) | 0 0% | 5 55.6% | 0 0% | 2 33.3% | 1 33.3% | 4 44.4% | 0.243 |
| Complication (n, %) | 1 16.7% | 3 33.3% | 0 0% | 2 33.3% | 2 66.7% | 4 44.4% | 0.372 |
| SSI (n, %) | 0 0% | 0 0% | 0 0% | 0 0% | 1 33.3% | 0 0% | **0.045** |
| Anastomotic leak (n, %) | 0 0% | 2 22.2% | 0 0% | 0 0% | 0 0% | 1 11.1% | 0.549 |
| Bleeding  (n, %) | 0 0% | 1 11.1% | 0 0% | 0 0% | 1 33.3% | 2 22.2% | 0.247 |
| Transfusion  (n, %) | 2 33.3% | 2 22.2% | 0 0% | 1 16.7% | 1 33.3% | 5 55.6% | 0.435 |
| Ileus (n, %) | 0 0% | 2 22.2% | 0 0% | 2 33 .3% | 0 0% | 2 22.2% | 0.549 |
| Clavien-Dindo (n, %)  0  1  2  3  4  5 | 2 33.3%  1 16.7%  3 50%  0 0%  0 0%  0 0% | 0 0%  4 44.4%  5 55.6%  0 0%  0 0%  0 0% | 3 100%  0 0%  0 0%  0 0%  0 0%  0 0% | 2 33.3%  2 33.3%  1 16.7%  0 0%  1 16.7%  0 0% | 1 33.3%  1 33.3%  1 33.3%  0 0%  0 0%  0 0% | 3 100%  0 0%  2 22.2%  1 11.1%  2 22.2%  1 11.1% | 0.366 |
| Reintervention (n, %) | 0 0% | 0 0% | 0 0% | 1 16.7% | 0 0% | 3 33.3% | 0.198 |
| Hb loss (mean, SD) | 2±1.1 | 1.6±1.2 | 2.3±0.7 | 1.6±0.9 | 1.9±0.3 | 1.2±0.5 | 0.478 |
| PCR  (mean, SD)  POD I  POD III  POD V | 56.4±30.2  136.7±92.2  139.6±9.1 | 65.9±38.8  147.6±82.2  98.8±60.3 | 40.7±16.8  65.5±30.3  49.1±53.6 | 64.7±44.9  96.9±53  54±49.7 | 66.7±41.7  170.4±181.9  132.1±117.3 | 61.8±32.2  144.3±111  148.9±73.9 | 0.925  0.684  0.413 |
| WBC  (mean, SD)  POD I  POD III | 18.33±22.68  7.45±2.69 | 10.68±6.176.79±1.36 | 9.67±0.41657.96±3.51 | 10.32±2.588.2±3.61 | 9.33±0.94  7.38±1.75 | 10.7±2.42  9.58±4.34 | 0.660  0.643 |
| PCT  (mean, SD)  POD III  POD V | 0.45±0.58  2.78±0.0 | 1.28±2  0.43±0.53 | 0.57±0.44  0.51±0.28 | 0.44±0.64  0.41±0.46 | 0.14±0.13  0.1±0.06 | 1.35±2.24  3.5±4.05 | 0.773  0.384 |

POD, post-operative day; CV, urinary catheter; NGT, naso-gastric tube; LOS, length of stay; pLOS, prolonged length of stay; SSI, surgical site infection; WBC, white blood count; CRP, C-reactive protein, PCT, procalcitonin.
